# Two-pore channels in MR1-dependent presentation of *Mycobacterium tuberculosis* infection

**DOI:** 10.1371/journal.ppat.1013342

**Published:** 2025-08-04

**Authors:** Elham Karamooz, Se-Jin Kim, Jessie C. Peterson, Allison E. Tammen, Shogo Soma, Aviva C.R. Soll, Erin W. Meermeier, Sharon Khuzwayo, David M. Lewinsohn

**Affiliations:** 1 Portland VA Medical Center, Portland, Oregon United States of America; 2 Pulmonary & Critical Care Medicine, Oregon Health & Science UniversityPortland, Oregon, United States of America; 3 Molecular Microbiology and Immunology, Oregon Health & Science UniversityPortland, Oregon, United States of America; 4 Department of Immunology, Mayo Clinic, Scottsdale, Arizona, United States of America; 5 Cape Town HVTN Immunology Laboratory, Hutchinson Centre Research Institute of South Africa, Cape Town, South Africa; New Jersey Medical School, UNITED STATES OF AMERICA

## Abstract

MR1 is a ubiquitously expressed MHC-Ib molecule that presents microbial metabolites to MR1-restricted T cells, but there are differences in the antigen presentation pathway of an intracellular microbe compared to exogenously delivered antigen. We have shown the importance of endosomal trafficking proteins in MR1-dependent presentation of *Mycobacterium tuberculosis* (Mtb) infection. Two pore channels (TPCs) are endosomal calcium channels that regulate endosomal trafficking. Due to their location on endosomes, we hypothesized that TPCs could be required for MR1-dependent presentation of antigens derived from the intracellular microbe Mtb. We found that TPC1 is critical for the presentation of Mtb infection by MR1; inhibition of TPCs had no effect on MR1 presentation of exogenously delivered antigens, HLA-B presentation, or HLA-II presentation. Finally, we found that the calcium-sensitive trafficking protein Synaptotagmin 7 was also key in the presentation of Mtb infection by MR1. TPC1 and Synaptotagmin 7 may be part of an endosomal pathway by which MR1 can sample intracellular mycobacterial infections.

## Author summary

MAIT cells are highly prevalent T cells that detect antigens presented on MR1. MR1 presents antigens derived from riboflavin biosynthesis. Since humans do not make riboflavin, MR1 is poised to detect the intracellular environment for non-self, small ligands. The mechanisms by which MR1 samples the intracellular environment for microbes are not well understood. We have previously found that endosomal trafficking proteins are important for MR1 presentation of *Mycobacterium tuberculosis* (Mtb) infection. To expand on those findings, we investigated the role of two-pore calcium channels (TPCs) and found that TPC1 is important for presentation of intracellular Mtb. Importantly, TPC1 played no role in MR1 presentation of exogenously delivered antigens. Furthermore, we found that the calcium-sensitive endosomal trafficking protein Synaptotagmin 7 also plays a role in the presentation of Mtb. Our results suggest that endosomal calcium signaling and calcium-sensitive trafficking proteins are necessary for MR1 presentation of intracellular microbes.

## Introduction

Mucosal Associated Invariant T cells (MAITs) are an abundant class of CD8 + T cells restricted by MR1 with a high prevalence in the peripheral blood as well as certain organs such as the lungs [1–3]. They detect a variety of pathogens, including *Mycobacterium tuberculosis* (Mtb), and produce proinflammatory cytokines in response to these microbes [1,2]. Unlike MHC-Ia molecules, which display peptides, MR1 is an MHC-Ib molecule that displays small microbial metabolites derived from riboflavin biosynthesis and other metabolic pathways [4–6]. Our previous work established the reliance of MR1-dependent presentation of Mtb infection on endosomal trafficking [7]. We also described differences between MR1 presentation of Mtb infection, an intracellular microbe, and exogenously delivered antigens [8]. Critically, the endosomal trafficking proteins identified in MR1-dependent antigen presentation play no role in HLA-B antigen presentation [7,8].

The human airway epithelial cell line BEAS-2B is very effective at presenting Mtb antigens to MAITs despite a lower infection efficiency compared to human dendritic cells (DCs) [9]. In DCs, Mtb interference with phagolysosome maturation results in a phagosome that is positive for the early endosome marker Rab5 [10]. In contrast, in BEAS-2B, Mtb resides in a late endosomal compartment defined by Rab7 and LAMP1 [9]. When MR1 tagged with GFP is overexpressed in these cells, the MR1 vesicles colocalize with Rab7 and LAMP1 by microscopy [7]. Despite the commonality of endosomal markers between the MR1 and Mtb compartment in BEAS-2B, it is unclear whether MR1 is physically present in the Mtb compartment.

Calcium signaling in T cells is necessary for cytokine release and proliferation but its role in antigen presentation is less clear [11]. Elevation of the intracellular calcium concentration can be diffuse or local. Late endosomes are rich in intracellular calcium and local calcium release regulates their trafficking [12,13]. This calcium release is governed by endosomal calcium channels, which include the mucolipins and two-pore channels (TPCs) [11,13]. Inhibition of TPCs leads to impaired endosomal trafficking, which has implications for the control of intracellular infections; for example, in a mouse model of Ebola virus infection, pharmacologic inhibition of TPCs decreased infectivity and improved survival by preventing the trafficking of the virus in the infected cell [14]. As TPCs are important for endosomal trafficking of viruses, we explored the role of TPCs and how intracellular calcium contributes to sampling the intracellular environment for Mtb.

## Results

**Fig 1:**
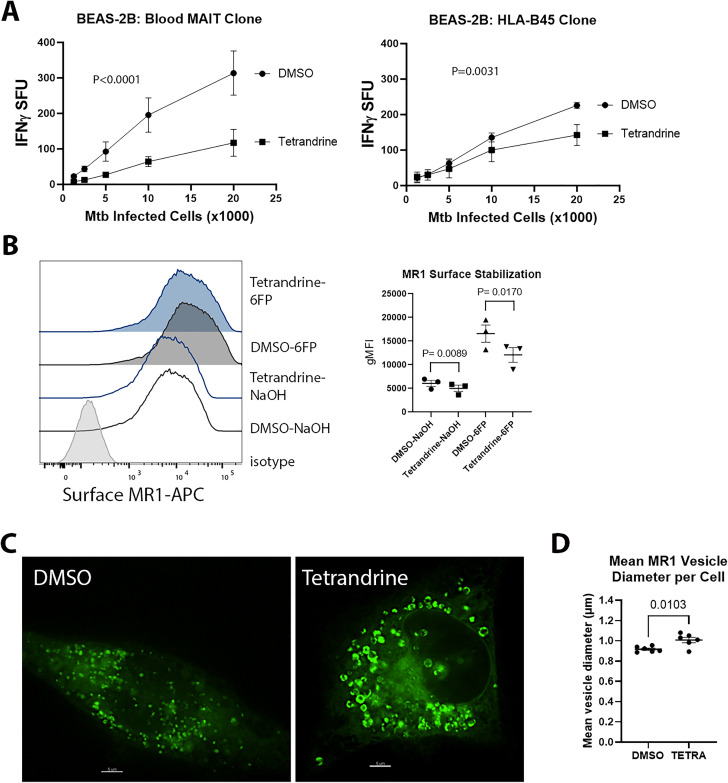
Tetrandrine decreases Mtb antigen presentation. **(A)** IFN-γ ELISpot assays in BEAS-2B infected with Mtb and treated with 10µM tetrandrine or DMSO. Cells were incubated with MAITs (left) or HLA-B45-restricted (right) T cell clones. Mean values from technical replicates were pooled from 3 independent experiments (mean and SEM graphed) and nonlinear regression analysis performed. **(B)** Effect of tetrandrine on MR1 surface stabilization in BEAS-2B transduced with BEAS-2B expressing doxycycline inducible MR1-GFP (TET-MR1GFP) treated with 100µM 6-FP or 0.01M NaOH. Representative histogram (left) and geometric MFI (gMFI) from 3 independent experiments (right). Mean difference in gMFI is 1049 for NaOH group and 4494 for 6-FP. P values from paired, two-tailed *t* tests. **(C)** Effect of tetrandrine on MR1-GFP vesicles in BEAS-2B transduced with TET-MR1GFP. Representative images from 3 independent experiments. A single Z-stack is shown. Scale bars represent 5µm. **(D)** Mean MR1 vesicle diameter with tetrandrine treatment. BEAS-2B transduced with TET-MR1GFP and induced with doxycycline were treated with 10µM tetrandrine versus DMSO. The next day, the cells were imaged and mean vesicle diameter per cell was calculated using Imaris. Significance was measured with an unpaired two-tailed *t* test.

First, we investigated whether calcium channel blockade with tetrandrine affects MR1-dependent presentation of Mtb infection. To measure antigen presentation, we used specific human T cell clones that produce IFN-γ in response to Mtb infected antigen presenting cells (APCs). We infected BEAS-2B with H37Rv Mtb with a multiplicity of infection (MOI) of 8. After 6 hours, cells were treated with 10µM tetrandrine or DMSO. After overnight treatment, the cells were incubated with a blood MAIT or an HLA-B45-restricted human T cell clone in an IFN-γ ELISpot assay. The blood MAIT cell clone is an MR1/5-OP-RU tetramer and TRAV1–2 positive clone that expresses CD26 and CD161, and had been isolated from a patient with latent tuberculosis [1,5,6]. When incubated with MR1 antigens in an A549 MR1 knockout cell line, it does not produce IFN-γ [8]. This T cell clone expresses CD8 and there is upregulation of CD69 when the MAIT cells are incubated with Mtb infected BEAS-2B (S1 Fig). We found that tetrandrine decreased both MR1- and HLA-B45-restricted antigen presentation (Fig 1A). On the other hand, the calcium channel blocker nifedipine had no effect on antigen presentation (S2 Fig). The small molecule 6-formylpterin (6-FP) is an MR1 antagonist derived from the photodegradation of folic acid [4]. Although unable to activate MAITs, 6-FP is a potent MR1 ligand that is loaded in the endoplasmic reticulum and induces MR1 translocation to the cell surface [15,16]. Using BEAS-2B stably transduced with a doxycycline inducible MR1-GFP construct (TET-MR1GFP), we found that tetrandrine decreased MR1 surface stabilization after treatment with 6-FP compared to control (Figs 1B and S3). We also found that tetrandrine caused enlargement of MR1GFP vesicles (Fig 1C-D).

**Fig 2:**
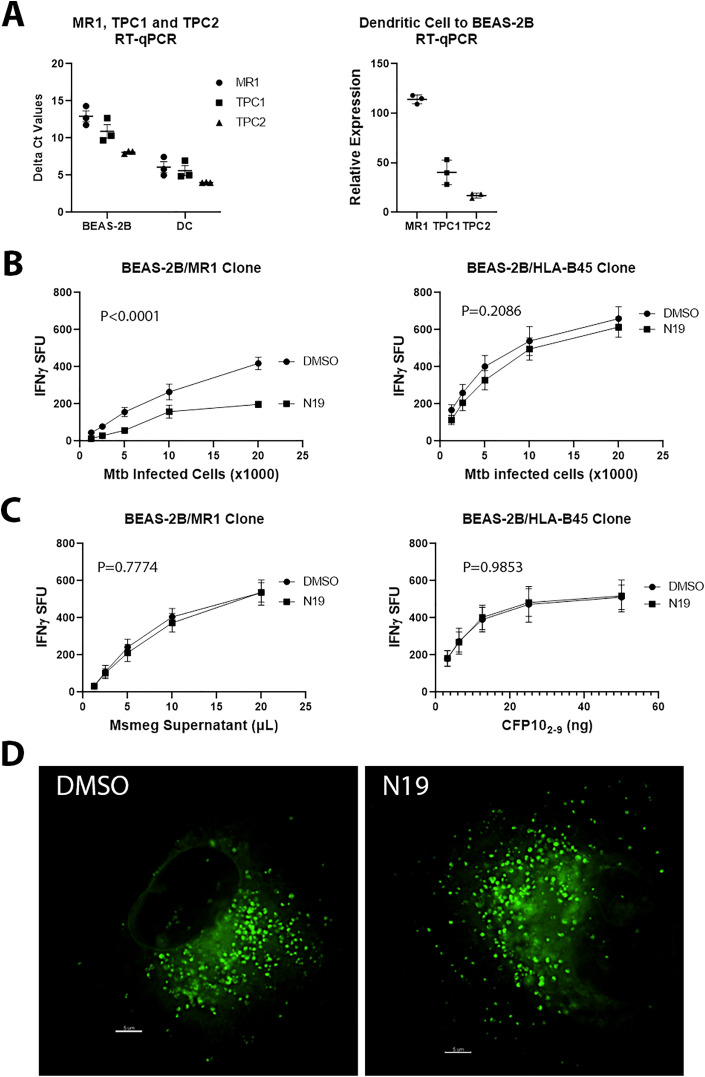
TPC inhibition specifically blocks MR1 presentation of Mtb infection. **(A)** Comparison of delta Ct values from RT-qPCR of MR1, TPC1, and TPC2 in BEAS-2B and DCs. Data were compared to GAPDH (left). Lower delta Ct values indicate higher expression. Relative expression of MR1, TPC1, and TPC2 in DCs were compared to BEAS-2B using RT-qPCR (right). **(B)** IFN-γ ELISpot assays of effect of N19 on MR1 and HLA-B45 antigen presentation of Mtb infection. (**C)** IFN-γ ELISpot assays of effect of N19 on MR1 and HLA-B45 presentation of exogenously delivered antigens. For (**B** and **C)**, mean values from technical replicates were pooled from 3 independent experiments (mean and SEM graphed) and nonlinear regression analysis performed. **(D)** Comparison of MR1-GFP vesicles in BEAS-2B expressing TET-MR1GFP treated with N19 or DMSO. Representative images from 3 independent experiments. A single Z-stack is shown. Scale bars represent 5µm.

The effects of tetrandrine on MR1- and HLA-B45-restricted antigen presentation suggested that the spectrum of activity of tetrandrine was too broad, therefore we sought to test the role of specific endosomal calcium channels in antigen presentation. We used RT-qPCR to determine the relative amounts of TPC1 and TPC2 in BEAS-2B and primary human DCs. We found that TPCs were expressed at levels similar to or greater than MR1 in both cell types and DCs had a higher expression of MR1, TPC1 and TPC2 compared to BEAS-2B (Fig 2A). TPCs release endosomal calcium in response to the ligand nicotinic acid adenine dinucleotide phosphate (NAADP), a process inhibited by the small molecule *trans*-Ned-19 (N19) [17,18]. To test the effect of N19 on antigen presentation, we treated BEAS-2B with 25µM N19 or DMSO 6 hours after Mtb infection and performed IFN-γ ELISpot assays. We found that N19 decreased MR1 presentation of Mtb infection but had no effect on HLA-B45 presentation (Fig 2B). N19 added simultaneously with Mtb infection maintained a reduction of MR1 antigen presentation (S4A Fig). Next, we used exogenously delivered antigens to test whether the effect of N19 was limited to Mtb. For MR1, we used filtered *Mycobacterium smegmatis* (Msmeg) supernatant and for the HLA-B45-restricted T cells, we used peptide CFP10_2–9_. We found that antigen presentation of exogenously delivered antigens was unaffected by N19 (Fig 2C). Since Mtb supernatant is poorly antigenic, we tested whether N19 affected the presentation of H37Rv cell wall, which is weakly antigenic [8]. We found no change in MR1 presentation of Mtb cell wall with N19 (S4B Fig). We also tested the effect of N19 on *Mycobacterium* avium infection, which is a nontuberculous mycobacteria (NTM), and found that N19 decrease MR1-dependent antigen presentation (S4C Fig). Microscopy of BEAS-2B transduced with TET-MR1GFP showed no perturbation of the vesicles by N19 (Fig 2D).

To determine if intracellular calcium chelation affects MR1 and HLA-B45 presentation of Mtb, we incubated BEAS-2B with BAPTA-AM and infected them simultaneously with Mtb. We found reductions in both MR1 and HLA-B45 antigen presentation, though the magnitude of the effect was larger for the Blood MAIT cell clone (S4D Fig). Finally, we tested the effect of N19 on Mtb presentation to a Lung MAIT clone derived from bronchoalveolar lavage and found reductions in IFN-γ (S5 Fig). These data indicate that TPC calcium release is important for MR1-mediated presentation of Mtb and NTM infection but not for HLA-B45 presentation of Mtb infection or exogenously delivered antigens.

**Fig 3:**
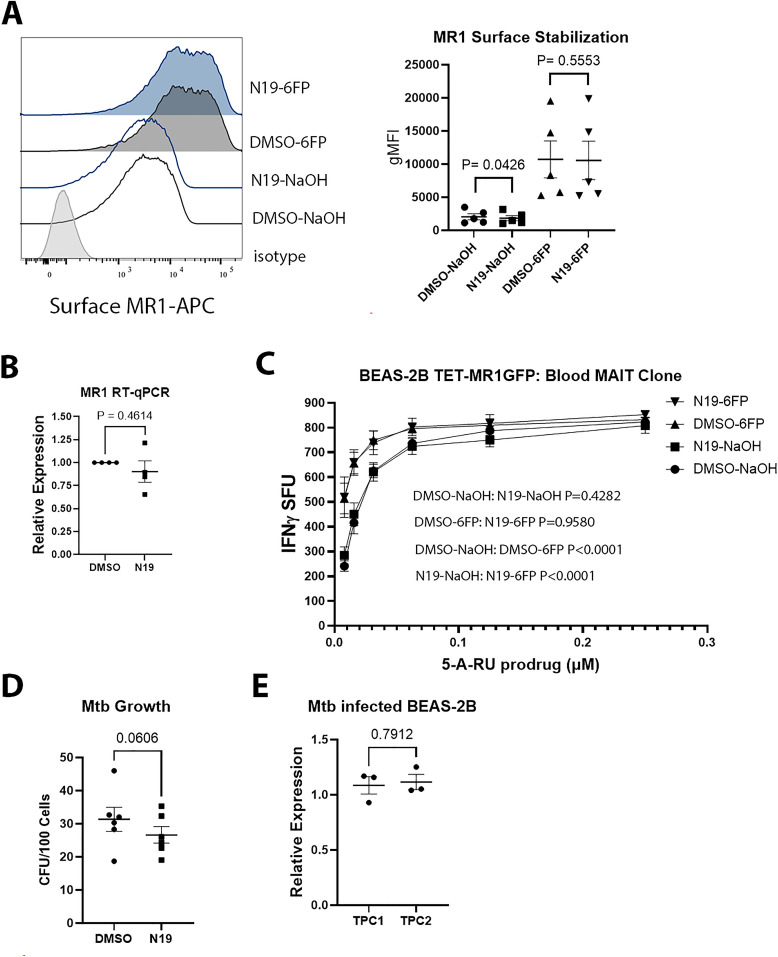
TPC inhibition does not affect MR1 surface stabilization or Mtb uptake. (**A**) Effect of N19 on MR1 surface stabilization in BEAS-2B expressing TET-MR1GFP treated with 100µM 6-FP versus 0.01M NaOH. Representative histogram (left) and gMFI from 5 independent experiments (right). Mean difference in gMFI is 223 for NaOH and 149 for 6-FP. P values from paired, two-tailed *t* tests. (**B**) RT-qPCR of MR1 transcripts in Mtb infected BEAS-2B treated with N19 versus DMSO. Data from 4 independent experiments plotted with mean and SEM. P value from paired, two-tailed *t* test. (**C**) Effect of N19 on presentation of 5-A-RU prodrug and 6-FP boosting in an IFN-γ ELISpot assay. Mean values from technical replicates were pooled from 3 independent experiments (mean and SEM graphed) and nonlinear regression analysis performed. (**D**) Colony forming units (CFU) from Mtb infected BEAS-2B treated with N19 versus DMSO. Mean from triplicates plotted from 3 independent experiments with SEM. P value from paired, two-tailed *t* test. (**E**) RT-qPCR of TPC1 and TPC2 on Mtb infected BEAS-2B. BEAS-2B were infected with H37Rv Mtb at an MOI of 8 and RT-qPCR was done on these cells and compared to uninfected BEAS-2B. Data were pooled from 3 independent experiments and an unpaired two-tailed *t* test performed.

To determine if N19 impacted MR1 surface stabilization, we performed flow cytometry on TET-MR1GFP cells treated with 6-FP. We found that N19 had no effect on MR1 surface stabilization after addition of 6-FP, though there was a small effect on overall surface MR1 without 6-FP treatment (Fig 3A). These data indicate that N19 does not interfere with MR1 loading in the ER or the ability of the 6-FP loaded MR1 to reach the cell surface. To determine whether N19 caused a change in MR1 transcripts in Mtb infected cells, we performed RT-qPCR on Mtb infected cells that were treated with N19 versus DMSO (Fig 3B). We found no difference in MR1 transcripts between the two conditions, indicating that the effect of N19 on Mtb infected cells was not due to changes in MR1 mRNA levels. In addition, N19 causes no significant changes in BEAS-2B viability (S6A-B Fig).

The most potent MR1 ligand is 5-OP-RU, a product of 5-A-RU and methylglyoxal [19]. Although 6-FP does not activate MAITs, pretreatment of APCs with 6-FP boosts MAIT responses to exogenously delivered antigens, including 5-OP-RU [8]. Because 5-A-RU is unstable, a 5-A-RU prodrug was developed that requires enzymatic cleavage in acidic endosomes to form 5-A-RU, where it is loaded onto MR1 in recycling endosomes [20]. To test whether N19 affected 6-FP mediated boosting of the 5-A-RU prodrug, we treated BEAS-2B expressing MR1GFP with N19 or DMSO and 6-FP or 0.01M NaOH. The next day, the cells were used in an IFN-γ ELISpot assay and additional N19 was added into the ELISpot wells so that N19 would be present with the 5-A-RU prodrug and the MAIT cells. We found that N19 had no effect on 5-A-RU prodrug presentation or the boosting effect from 6-FP pretreatment (Fig 3C). Because N19 was incubated with MAIT cells in the ELISpot assay, these data also show that N19 has no direct effect on MAITs or their ability to produce IFN-γ. As an orthogonal approach to determining whether N19 alters endosomal pH, we used pHrodo dextran green and found only a small increase in acidity, whereas tetrandrine had a larger effect (S6C-D Fig). Altogether, these data show that N19 causes only minor pH changes in endosomes.

The effect of N19 on MR1 presentation of Mtb infection raised the possibility that N19 could affect Mtb uptake or Mtb viability. Either mechanism would lead to a decrease in the quantity of Mtb antigens available to MR1. Previous work showed that N19 can affect small and large particle uptake in short assays of up to 45 minutes [21]. While N19 did not affect the processing or presentation of Mtb derived CFP10 (Fig 2C), we sought to directly test whether N19 affected Mtb uptake or Mtb viability. We performed growth assays on Mtb infected BEAS-2B treated with N19 and found a mean difference of 4.72 Mtb colony forming units (CFU) per 100 cells, which was not statistically significant. (Fig 3D). These data are consistent with the Mtb/HLA-B45 assays, which showed no effect from N19. As additional controls, we tested whether Mtb infection altered expression of TPCs in BEAS-2Bs and found no biologically relevant increases in TPC transcripts compared to uninfected cells (Fig 3E).

**Fig 4:**
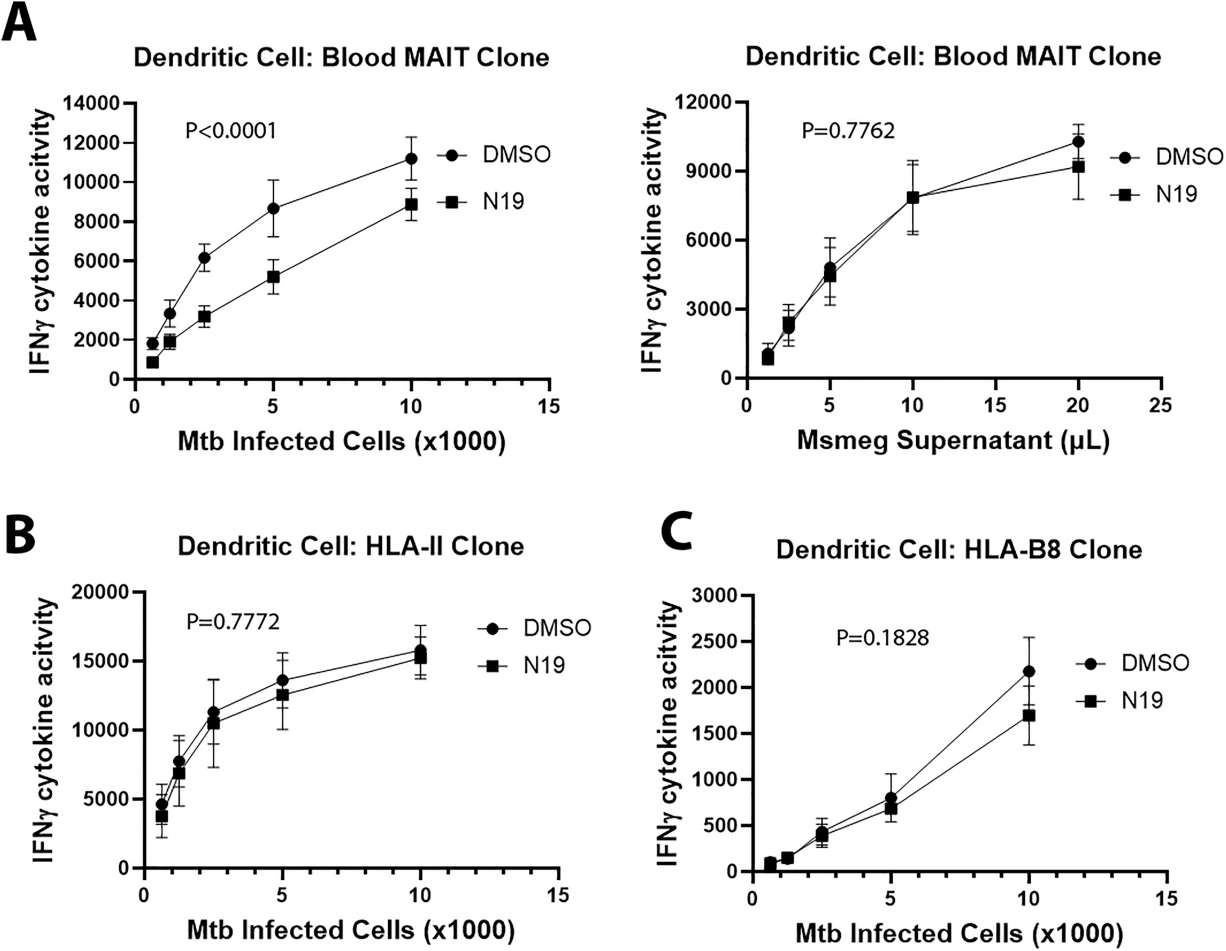
TPC inhibition blocks MR1 presentation of Mtb infection in DCs. **(A)** IFN-γ ELISpot assays of DCs treated with 100µM N19 and incubated with MAITs. Antigen presentation of Mtb infection (left) versus Msmeg supernatant (right). **(B)** IFN-γ ELISpot assays with HLA-II T cell clone and Mtb infected DCs treated with 100µM N19. **(C)** IFN-γ ELISpot assays with HLA-B8 T cell clone and Mtb infected DCs treated with 100µM N19. For **(A-C)**, mean values from technical replicates were pooled from 3 independent experiments (mean and SEM graphed) and nonlinear regression analysis performed.

Although BEAS-2B present Mtb to MAITs, BEAS-2B are not professional APCs and the mechanisms underlying myeloid presentation of Mtb infection likely differ from those of airway epithelial cells. To determine whether the effect of N19 was generalizable to professional APCs, we treated primary human DCs with 100µM N19 or DMSO 6 hours after Mtb infection and performed IFN-γ ELISpot assays. We found that N19 affected MR1-dependent presentation of Mtb infection in DCs (Fig 4A). There was no effect on MR1-dependent presentation of exogenously delivered antigens (Fig 4A). Since DCs have HLA-II, which has a well-established endosomal trafficking component, we characterized the effect of N19 on HLA-II presentation of Mtb infection using an HLA-II-restricted T cell clone that detects CFP10 [10]. An HLA-B8-restricted T cell clone was used as a class I control and has been characterized previously [10,22]. We found that N19 had no effect on HLA-II or HLA-B8 presentation in DCs (Fig 4B and 4C). These data show that in DCs, N19 specifically affects MR1 presentation of Mtb infection without impacting HLA-II, HLA-B8 or exogenously delivered antigen presentation.

**Fig 5:**
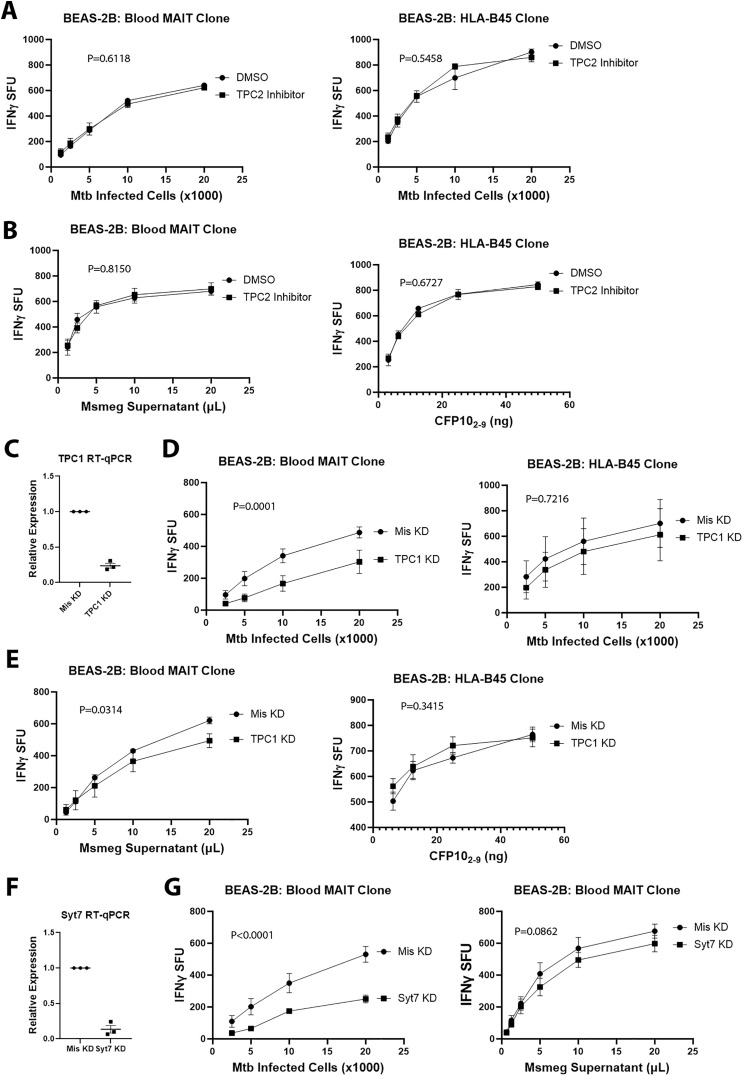
Knockdown of TPC1 and Synaptotagmin 7 specifically impair MR1 presentation of Mtb infection. **(A)** IFN-γ ELISpot assays measuring effect of 5µM YM201636 on MR1 and HLA-B45 presentation of Mtb. **(B)** IFN-γ ELISpot assays of BEAS-2B presentation of exogenously delivered antigen by MR1 and HLA-B45 after treatment with 5µM YM201636. For **(A and B)**, mean values from technical replicates were pooled from 3 independent experiments (mean and SEM graphed) and nonlinear regression analysis performed. **(C)** RT-qPCR of TPC1 siRNA knockdown from 3 independent experiments. Mean and SEM graphed. **(D)** IFN-γ ELISpot assays of Mtb infected TPC1 knockdown BEAS-2B using MR1- (left) and HLA-B45-restricted T cells (right). **(E)** IFN-γ ELISpot assays of MR1 and HLA-B45 presentation of exogenously delivered antigen by BEAS-2B after TPC1 knockdown. For **(D and E)**, mean values from technical replicates were pooled from 3 independent experiments (mean and SEM graphed) and nonlinear regression analysis performed. **(F)** RT-qPCR of Syt7 siRNA knockdown from 3 independent experiments. Mean and SEM graphed. **(G)** IFN-γ ELISpot assays of Syt7 knockdown BEAS-2B with Mtb infection (left) versus Msmeg supernatant (right). Mean values from technical replicates were pooled from 3 independent experiments (mean and SEM graphed) and nonlinear regression analysis performed.

In humans, there are two TPC proteins, TPC1 and TPC2 [12,13]. To confirm that the effect of N19 was due to inhibition of TPCs, we first used the TPC2 inhibitor YM201636 [23]. Inhibition of TPC2 in BEAS-2B had no effect on MR1 or HLA-B45 antigen presentation of Mtb or exogenously delivered antigens (Fig 5A and 5B). Since a TPC1 inhibitor is not available, we performed siRNA knockdown of TPC1 in BEAS-2B. Knockdown of TPC1 reduced TPC1 transcripts by 76% in 48 hours (Fig 5C). To determine the association between MR1 and TPC1, we performed fluorescence microscopy on BEAS-2B expressing MR1GFP and transfected with TPC1-RFP. We found that 44% of MR1 vesicles colocalize with TPC1 (S7A-B Fig). There was no increase in cell toxicity with TPC1 knockdown, nor any changes in total or surface MR1 (S7C-D Fig). Furthermore, TPC1 knockdown had no effect on MR1 colocalization with LAMP1 (S7E Fig). Functionally, TPC1 knockdown resulted in a significant reduction in MR1-mediated presentation of Mtb infection and a small effect on MR1 presentation of exogenously delivered antigens (Fig 5D and 5E). However, there was no significant effect on HLA-B45 presentation of Mtb or exogenously delivered antigen (Fig 5D and 5E). Although it is possible that N19 is altering the Mtb metabolome, thus affecting MR1-dependent presentation of Mtb infection, TPC1 knockdown by siRNA is directed solely at the APC, thus it is unlikely that TPC1 knockdown is affecting the Mtb metabolome.

Since TPC-mediated calcium release facilitates endosomal trafficking, and TPC1 is associated with early, recycling and late endosomes [24,25], we hypothesized that calcium-sensitive endosomal trafficking proteins play a role in MR1 presentation of Mtb infection. Synaptotagmin 7 (Syt7) is a calcium-sensitive endosomal trafficking protein implicated in LAMP1 delivery to phagosomes and translocation of MHC-II to the plasma membrane [26,27]. To test the role of Syt7 in MR1-dependent antigen presentation, we knocked down Syt7 using siRNA, resulting in an 86% reduction in Syt7 transcripts (Fig 5F). Functionally, Syt7 knockdown affected MR1-dependent presentation of Mtb infection without a significant effect on MR1 presentation of exogenously delivered antigens (Fig 5G). Taken with the TPC1 knockdown experiments, these data show that TPC1 and Syt7 knockdown have MR1-Mtb specific effects consistent with N19 treatment.

## Discussion

McWilliam and colleagues established the importance of the endoplasmic reticulum (ER) in MR1 antigen presentation [16]. In this model, a preformed pool of MR1 is retained in the ER. After acquiring a ligand that forms a Schiff base with lysine 43 of MR1, MR1 associates with β_2_M and translocates to the cell surface [16]. This has been demonstrated with exogenously added ligands such as Acetyl-6-FP and 5-OP-RU. In the setting of *Salmonella enterica* serovar Typhimurium infection, MR1-dependent antigen presentation was substantially reduced by Brefeldin A, which prevents protein egress from the ER [16]. Follow-up studies using a fluorescent MR1 ligand confirmed that the loading occurred in the ER [28]. In contrast, our studies focus on the mechanism of MR1 presentation of antigens derived from Mtb infection. We previously found that certain endosomal trafficking proteins played a role in MR1 antigen presentation and elucidated differences in antigen presentation pathways between intracellular Mtb infection and exogenously delivered mycobacterial antigens [7,8]. Specifically, we showed that pretreatment with the inhibitory ligand 6-FP boosted presentation of exogenously delivered antigens but had no such effect with Mtb infection [8]. Furthermore, we found that Syntaxin 4 knockdown inhibited exogenously delivered antigen presentation by MR1 but had no effect on Mtb presentation [8]. These results implied that the MR1 antigen presentation pathway differed for Mtb compared to exogenously delivered antigens.

In this study, we expand on our earlier work and show TPC1 and Syt7 are important in MR1 presentation of intracellular Mtb infection. First, we show that treatment with calcium channel blocker tetrandrine decreased both MR1- and HLA-B45-dependent presentation of Mtb infection. Next, we investigated endosomal calcium channels given our prior work on the importance of endosomal trafficking in MR1. Since late endosomes are rich in calcium and express TPCs [12], we targeted TPCs using N19, which inhibits NAADP-mediated calcium release from TPCs [18]. We found a significant and specific decrease in Mtb antigen presentation to MAITs in both human airway epithelial cells and primary human DCs. There was no effect on exogenously delivered antigen presentation, further supporting that the mechanisms of MR1 presentation of Mtb differs from that of exogenously delivered antigens. We also found that N19 decreases MR1-dependent presentation of the NTM *Mycobacterium avium*. It is unlikely that the mechanism of N19 inhibition of MR1-Mtb presentation stems from blocking of acidification or recycling of the endosome because N19 had no effect on the 5-A-RU prodrug, which requires an acidic compartment for cleavage into 5-A-RU and recycling endosomes for presentation [20]. Although the inhibition of MR1 presentation of Mtb infection by N19 is partial, we hypothesize that there are redundant proteins that may compensate for pharmacologic inhibition.

Previous work indicated that TPCs are important for the phagocytic function of murine bone marrow-derived macrophages [21]. In that work, uptake of silica beads, *Mycobacterium smegmatis* and an attenuated form of *Mycobacterium bovis* (BCG) were used to measure phagocytic function. However, uptake was measured at a maximum of 120 minutes. Given those results, we performed orthogonal assays to determine whether our findings were due to impaired Mtb uptake or unexpected toxicity from N19. First, we used an HLA-B45 restricted T cell clone that detects CFP10_2–9_ from Mtb. The results showed no significant effect of N19 on HLA-B45 antigen presentation, indicating a similar amount of internalized Mtb antigen between the groups. Additionally, we performed growth assays between the N19 and control groups and found no significant difference in the number of viable Mtb between conditions. These data argue that the results with N19 do not reflect impaired uptake or mycobacterial viability.

**Fig 6:**
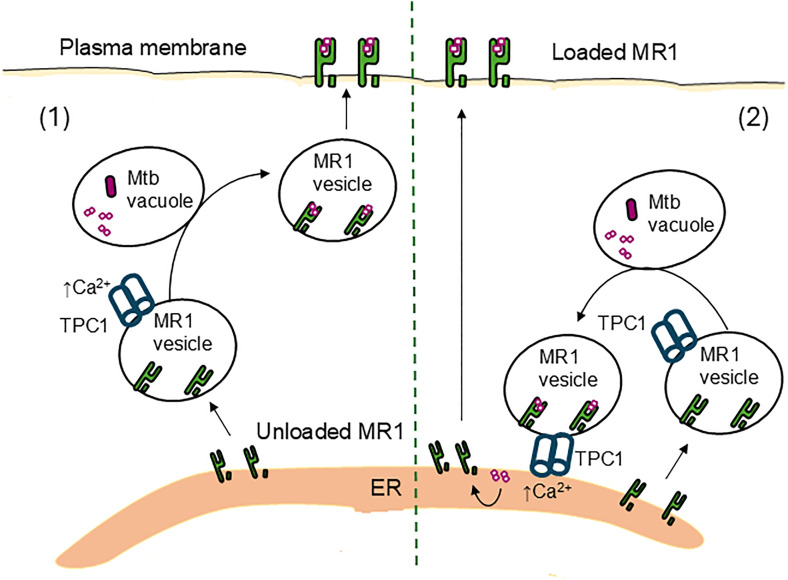
Model of how TPC1s mediate MR1-dependent presentation of Mtb infection. There are 2 possible mechanisms for TPC1 mediating MR1 presentation of Mtb infection. [1] MR1 moves from the endoplasmic reticulum (ER) into an MR1 vesicle with TPC1. Calcium release from TPC1 allows MR1 to contact the Mtb containing vacuole, where it is loaded with Mtb antigens. Afterwards, loaded MR1 traffics to the cell surface. [2] MR1 vesicles capture Mtb antigens from the Mtb containing vacuole. Then using TPC1, they form ER-endosome contact sites, which transfer antigen to unloaded MR1 in the ER. Loaded MR1 then traffics to the cell surface.

The results obtained with N19 were confirmed with TPC1 knockdown. We then evaluated the role of the calcium-sensitive endosomal trafficking protein Syt7 and found that it is also key to MR1-dependent presentation of Mtb infection. These knockdowns are directed at the wild-type BEAS-2B, with only native MR1 expression, and are unlikely to affect the mycobacterial metabolome. Mechanistically, there are different plausible roles for TPC1 and Syt7 in MR1-dependent antigen presentation. TPC1 is expressed in different organs, including in the lungs, and colocalizes with recycling endosomes, endolysosomes and late endosomes [24,25]. Therefore, TPC1 could be responsible for local endosomal calcium release that activates Syt7. However, in HeLa cells and primary human fibroblasts, TPC1 is required for the formation of late endosome-ER contact sites [29]; both N19 and TPC1 knockdown reduced the number of contacts between late endosomes and the ER [29]. Therefore, it is possible that TPC1-mediated endosome-ER contact sites could deliver MR1 antigens derived from Mtb infection to the ER, which would then allow MR1 egress from the ER and translocation to the cell surface (Fig 6).

Regarding the mechanism of Syt7 in MR1-dependent antigen presentation, at least two possibilities exist. First, in murine bone marrow derived macrophages, Syt7 functions in the early recruitment of LAMP1 to the phagosome [26]. In human DCs and BEAS-2B, the percentage of Mtb positive for LAMP1 was 90% and 58%-80% (depending on the time point), respectively [9,30]. Thus, it is possible that Syt7 knockdown interferes with LAMP1–and possibly MR1– delivery to the Mtb compartment and that this is necessary for MR1 presentation of Mtb infection. A second possibility is that Syt7 shuttles loaded MR1 to the cell surface. This is mechanism is based on data from Syt7 knockout mice, where DCs had decreased MHC-II surface expression [26]. Given that MR1 antigens are small molecules and produced in small quantities by Mtb, tracking the how these antigens interact with TPC1 and Syt7 is a significant challenge. Hence, the exact mechanism by which TPC1 and Syt7 function in MR1-dependent presentation of Mtb remains unknown, but these pathways could be important for other intracellular microbes besides mycobacteria.

In conclusion, we have identified a pathway for MR1-dependent presentation of Mtb infection that utilizes the endosomal calcium channel TPC1 and the calcium-sensitive trafficking protein Syt7. This pathway is specific to MR1 and has no role in HLA-B or HLA-II presentation. Although the exact mechanism is still unknown, this pathway may represent a way in which MR1 samples the intracellular environment for minute quantities of antigen derived from Mtb infection.

## Materials and methods

### Bacteria and cells

*Mycobacterium tuberculosis* (Mtb) H37Rv (ATCC) and *Mycobacterium* avium Chester (ATCC) were grown in Middlebrook 7H9 broth supplemented with Middlebrook ADC, 0.05% Tween-80, and 0.5% glycerol. The bacteria were passaged 10–20 times through a tuberculin syringe before infection. Multiplicity of infection of 8 was used for all Mtb experiments and 22 for *Mycobacterium avium*. Mtb growth assays were performed by lysing 200,000 infected BEAS-2B in ultrapure water. Serial dilutions were performed with PBS + 0.05% Tween 80 and lysates were plated on 7H10 plates supplemented with glycerol and Middlebrook ADC. Plates were incubated at 37 C and 5% CO_2_. *Mycobacterium smegmatis* (Msmeg) supernatant was passed through a 0.22 µm filter and concentrated across a 10kDa Amicon filter (Millipore Sigma). The supernatant was aliquoted and stored at -80C. All experiments with Mtb were done in a Biosafety Level 3 laboratory. Waste was decontaminated in 3% Wescodyne and autoclaved. All other experiments were performed in a Biosafety Level 2 laboratory.

BEAS-2B (ATCC) and BEAS-2B transduced with TET-MR1GFP [31] were cultured in DMEM (Gibco) supplemented with L-glutamine and 10% heat inactivated fetal bovine serum (FBS). Human dendritic cells (DCs) from donor D454 were prepared from peripheral blood mononuclear cells [1,32]. Briefly, PBMC were resuspended in 10% heat inactivated human serum in RPMI (Gibco) supplemented with L-glutamine (Gibco), gentamicin (Gibco) and DNAse (Roche), and placed in a T-75 flask and allowed to adhere at 37C with 5% CO2. After 1 hour, the flask was gently rocked and nonadherent cells removed. The cells were treated with 300ng GM-CSF (Sanofi) and 300ng IL-4 (R&D Systems). The flask was irradiated with 3000cgray and DCs were used on day 5. D454 DCs were used for all DC experiments. The following human T cell clones were used: Blood MAIT D426-G11 [1,5,6], Lung MAIT D1004-A3 [33], HLA-B45-restricted (D466-A10, minimal epitope CFP10_2–9_) [34], HLA-B8-restricted (D480-F6, minimal epitope CFP10_3–11_) [10,22], and HLA-II restricted (D454-E12, detects CFP10_25–39_) [10].

### Reagents and antibodies

Tetrandrine (Selleck Chemicals) was obtained as a 10mM stock in DMSO and used at 10µM. *Trans*-Ned-19 (N19, Cayman Chemical) was resuspended to 2.5mg/mL in DMSO and used at 25µM for BEAS-2B and 100µM in DCs. YM-201636 (MedChemExpress) was obtained as a 10mM stock in DMSO and used at 5µM. 6-formylpterin (Schirck’s Laboratories) was resuspended at 1mg/mL in 0.01M NaOH and used at 100µM. Doxycycline (Sigma-Aldrich) was suspended at 2mg/mL in sterile water and used at 2µg/mL. 5-A-RU prodrug (5-A-RU-PABC-Val-Cit-Fmoc, MedChemExpress) was resuspended in DMSO at 10mM. BAPTA-AM (Caymen Chemical) was used at 10 µM. pHrodo Dextran Green (ThermoFisher) was used at 33µg/mL. H37Rv cell wall (BEI Resources) was diluted in PBS to 5μg/mL and used at 25μg. Phytohemagglutinin (PHA, Sigma-Aldrich) was resuspended to 10mg/mL in 10% human serum in RPMI (Gibco) supplemented with L-glutamine and gentamicin. Paraformaldehyde (Electron Microscopy Sciences) was obtained as a 16% stock and diluted to a 4% working stock using phosphate buffered saline (Corning). Anti-MR1 mouse monoclonal antibody APC (Clone 26.5; BioLegend) and isotype control antibody mouse IgG2a (BioLegend) were used at 1:166. FBS (GeminiBio) was heat inactivated at 56C for 45 minutes. Human serum was obtained from donors and heat inactivated.

### ELISpot assays

ELISpot assays were done using 96 well MSHA plates (Merck Millipore) and coated with an antibody against IFN-γ (Mabtech). Antigen presenting cells were harvested using 10% human serum in RPMI (Gibco) supplemented with L-glutamine and gentamicin. The cells were resuspended to 2e5 for Mtb infected BEAS-2B and 1e5 for DCs and uninfected BEAS-2B. The cells were plated at 100µL/well in the MSHA plates in duplicate. For Mtb experiments with tetrandrine, N19, or YM-201636, cells were treated with drug 6 hours after Mtb infection. N19 experiments in S4 and S5 Figs were done with N19 and Mtb added simultaneously. For all Mtb infected APCs, serial dilutions were performed by counting cells on a hemocytometer with trypan blue staining. For uninfected APCs, the APCs were kept constant and Msmeg supernatant or peptide was added as a serial dilution with the final volumes kept constant. PHA was used as a positive control for all ELISpot assays (1µg/well). After 1 hour, T cells were added at 1e4 cells/well. After overnight incubation at 37C and 5% CO_2_, the plate was developed with ALP antibody (Mabtech). For the Lung MAIT experiments, T cells were added at 2e4 cells/well, IFN-γ spot forming units (SFU) or IFN-γ cytokine activity were measured on an AID ELISpot reader. The mean of technical replicates was used to pool data from different experiments.

ELISpot assays using the 5-A-RU prodrug were done with BEAS-2B stably transduced with TET-MR1GFP. The cells were cultured in a 6 well plate and MR1GFP was induced with 2µg/mL doxycycline. The next day, the cells were treated with 25µM N19 versus DMSO and 100µM 6-FP versus 0.01M NaOH. The next day, the cells were harvested and resuspended to 1e5 cells/mL and re-treated with N19 versus DMSO, then plated at 100µL/well (1e4 cells) in duplicate (final concentration of N19 was 25µM in the ELISpot well). 5-A-RU prodrug was diluted to 1µM in 10% human serum and RPMI supplemented with L-glutamine and gentamicin. 5-A-RU prodrug was added at 50µL/well and serial dilutions performed (starting concentration in the wells was 0.25µM). After 1 hour, T cells were added at 1e4 cells/well (50µL/well) resulting in a final volume of 200µL/well.

### Flow cytometry

BEAS-2B transduced with TET-MR1GFP were induced with doxycycline in a 6 well plate (Corning). The next day, the cells were treated with drug versus DMSO as well as 100µM 6-FP versus 0.01M NaOH. The next day, the cells were stained for surface MR1 versus isotype control in 2% human serum, 2% goat serum, and 0.5% FBS on ice. After 40 minutes, the cells were washed, fixed in 1% PFA and analyzed with a BD LSR II flow cytometer. All analyses were performed using FlowJo software (BD). For TPC1 knockdown TET-MR1GFP BEAS-2B, cells were induced with doxycycline and total and surface MR1 was measured without 6-FP.

### siRNA knockdown

BEAS-2B plated in 6-well tissue culture plates (Corning) at 70% confluency were transfected with 50nM Silencer Select siRNA (Missense Negative Control #1, TPCN1 s28727 or SYT7 s17292) using HiPerFect (Qiagen). Knockdown was done for 48 hours. For functional assays, the knockdown cells were infected with Mtb or left uninfected for exogenously delivered antigens, and used the following day in an IFN-γ ELISpot assay.

### RNA isolation, cDNA synthesis and RT-qPCR analysis

Total RNA was isolated using RNeasy Mini Kit (Qiagen). cDNA was synthesized using a High Capacity cDNA Reverse Transcription Kit (ThermoFisher Scientific). RT-qPCR was performed using TaqMan Universal PCR Master Mix (ThermoFisher Scientific) on a Step One Plus Real-Time PCR System (Applied Biosystems). FAM-MGB TaqMan Gene Expression Assays for all targets were obtained from ThermoFisher Scientific. Reactions were run in triplicate and data normalized to GAPDH. Expression levels were determined using the 2^-ΔΔCT^ method.

### Microscopy

BEAS-2B transduced with TET-MR1GFP were plated into a 4-well 1.5 mm glass bottom chamber slides (Nunc) and incubated at 37C and 5% CO2. After 24 hours of doxycycline treatment, the cells were treated with test drug (tetrandrine or N19) versus DMSO control. The next day, the cells were imaged live on a DeltaVision Widefield Deconvolution microscope with a 60x objective (NA 1.42) and a Nikon Coolsnap ES2 HQ. Each image was acquired as Z-stacks (0.5µm) from the top of the cell through the bottom of the cell in a 1024 × 1024 format. Images were processed on Imaris (Bitplane). Vesicle diameter was measured on Imaris (Bitplane).

TPCN1 isoform 2 was designed with a C terminus RFP tag (ThermoFisher Scientific). The construct (TPC1-RFP) was flanked by EcoRI and KpnI and was cloned into our previously described PCI plasmid [7]. TPC1-RFP was transfected into BEAS-2B transduced with TET-MR1GFP using an Amaxa Nucelofector and Kit T solution (Lonza). Each transfection reaction was done with 1e6 cells and 1.5μg of TPC1-RFP plasmid. Transfected cells were plated into chamber slides and MR1GFP was induced with 2µg/mL doxycycline. The next day, the cells were imaged as described above. Colocalization was measured on Imaris (Bitplane).

For MR1 and LAMP1 colocalization experiments, TPC1 was knocked down in TET-MR1GFP BEAS-2B. MR1 was induced with doxycycline and the cells were treated with CellLight Lysosomes-RFP (ThermoFisher). The cells were imaged live on a Nikon Spinning Disk microscope with a 100x objective (NA 1.49). Colocalization was measured on Imaris (Bitplane).

### Ethics statement

This study was conducted according to the principles expressed in the Declaration of Helsinki. Study participants, protocols and consent forms were approved by Oregon Health & Science University Institutional Review Board (IRB00000186). Written and informed consent was obtained from all donors.

### Statistical analysis

Data were analyzed with GraphPad Prism 9.5. All ELISpot assays were plated with technical replicates. Mean data from technical replicates were pooled from 3 independent experiments. Nonlinear regression was done using agonist vs. response (3 parameters) and a bottom constraint set to 0. For flow cytometry, data were pooled from 3 independent experiments. For all *t* tests, a two-tailed analysis was performed.

## Supporting information

S1 Raw Data Raw data for all experiments. (XLSX)

S1 Fig Mtb infection of BEAS-2B increases CD69 on MAIT cells in co-culture. (**A)** Gating strategy on MAIT cells that are CD8 high. (**B**) CD69 gMFI pooled from two independent experiments. Significance measured by a paired two tailed *t* test. P = 0.0007.
(TIF)

S2 Fig Nifedipine has no effect on Mtb antigen presentation. (**A**) IFN-γ ELISpot assays of BEAS-2B infected with Mtb and treated with 50µM Nifedipine or DMSO. Cells were incubated with MAITs (left) or HLA-B45-restricted (right) T cell clones. Mean values from technical replicates were pooled from 4 independent experiments (mean and SEM graphed). (**B**) IFN-γ ELISpot assays of effect of Nifedipine on MR1 and HLA-B45 presentation of exogenous antigens. Mean values from technical replicates were pooled from 4 independent experiments (mean and SEM graphed).
(TIF)

S3 Fig Gating strategy for MR1 surface staining. (TIF)

S4 Fig Further characterization of N19 and calcium dependence. (**A**) N19 decreases MR1 presentation of Mtb when added at the same time as infection. 25µM N19 was added to BEAS-2Bs the same time as Mtb (MOI 8). The next day, the cells were incubated with a Blood MAIT clone as discussed in Fig 1. Pooled data from 3 independent experiments. P < 0.0001. (**B**) N19 has no effect on exogenously added Mtb cell wall. 25µg of Mtb cell wall was incubated with BEAS-2B treated with N19 versus DMSO and co-incubated with a Blood MAIT clone. Pooled data from 2 independent experiments. Significance measured by a paired two tailed *t* test. P = 0.15. (**C**) N19 decreases MR1 dependent presentation of nontuberculous mycobacteria (NTM). IFN-γ ELISpot assays of BEAS-2B infected with NTM (*Mycobacterium avium* Chester, ATCC) at an MOI of 22 and treated with 25µM N19 or DMSO. Cells were incubated with MAIT cell clones and IFN-γ spot forming units (SFU) measured. Mean values from technical replicates were pooled from 3 independent experiments (mean and SEM graphed). (**D**) Effect of the calcium chelator BAPTA-AM on MR1 presentation of Mtb. BEAS-2B were treated with 10µM BAPTA-AM versus DMSO. Mtb was added at the same time (MOI 8). Cells were incubated with a Blood MAIT clone versus an HLA-B45 restricted clone. Pooled data from 2 independent experiments. Left, P < 0.0001. Right, P = 0.0001.
(TIF)

S5 Fig Effect of N19 on a TRAV1–2 Lung MAIT clone. (**A**) Lung MAIT was isolated from BAL as described by Wong *et al* [33]. Flow cytometry characterizing Lung MAIT D1004-A3 as an MR1/5-OP-RU tetramer positive, TRAV1–2 + MAIT cell. (**B**) Effect of 25µM N19 versus DMSO on Mtb infected BEAS-2B incubating with 20,000 D1004-A3 clones. Pooled data from 3 independent experiments. P = 0.0043.
(TIF)

S6 Fig N19 live dead staining and pHrodo measurements. (**A**) Gating strategy for live dead staining of N19 and DMSO treated BEAS-2B. (**B**) Mean data plotted from technical replicates of live dead staining from 2 independent experiments. Significance measured with an unpaired *t* test. (**C**) Flow histogram of pHrodo dextran green of BEAS-2B treated with DMSO, N19 or tetrandrine. (**D**) Pooled data of pHrodo dextran green from 2 independent experiments. gMFI: DMSO = 585.5, N19 = 623.0, tetrandrine = 1117. Statistical significance measured with paired two tailed *t* test.
(TIF)

S7 Fig TPC1 microscopy and flow cytometry. (**A**) Microscopy of MR1-GFP and TPC1-RFP. BEAS-2B expressing TET-MR1GFP transfected with TPC1-RFP. MR1GFP was induced with doxycycline and cells imaged the next day. Nuclei were stained with Hoechst 33342. Images are representative of 30 different images from 3 independent experiments. Scale bars represent 5µm. (**B**) Quantification of percent of MR1 vesicles that localized with TPC1. Data are graphed as mean and standard deviation. Mean percentage is 44% with a standard deviation of 14.5%. (**C)** Live dead staining of TPC1 knockdown in BEAS-2B. 2 independent experiments performed. Statistical analysis performed with a paired *t* test. (**D**) Total (left) and surface (right) MR1 in TET-MR1GFP cells with TPC1 knocked down. Analysis as performed in (**C**). (**E**) LAMP1 colocalization with MR1 in TET-MR1GFP cells with TPC1 knock down. 10 cells imaged in each condition. Statistical analysis done by an unpaired *t* test.
(TIF)
