# Supplementary figures and images for: Two-pore channels in MR1-dependent presentation of Mycobacterium tuberculosis infection
Source: PLoS Pathog. 2025 Aug 4;21(8):e1013342. doi: 10.1371/journal.ppat.1013342 (PMC12331044; doi:10.1371/journal.ppat.1013342)

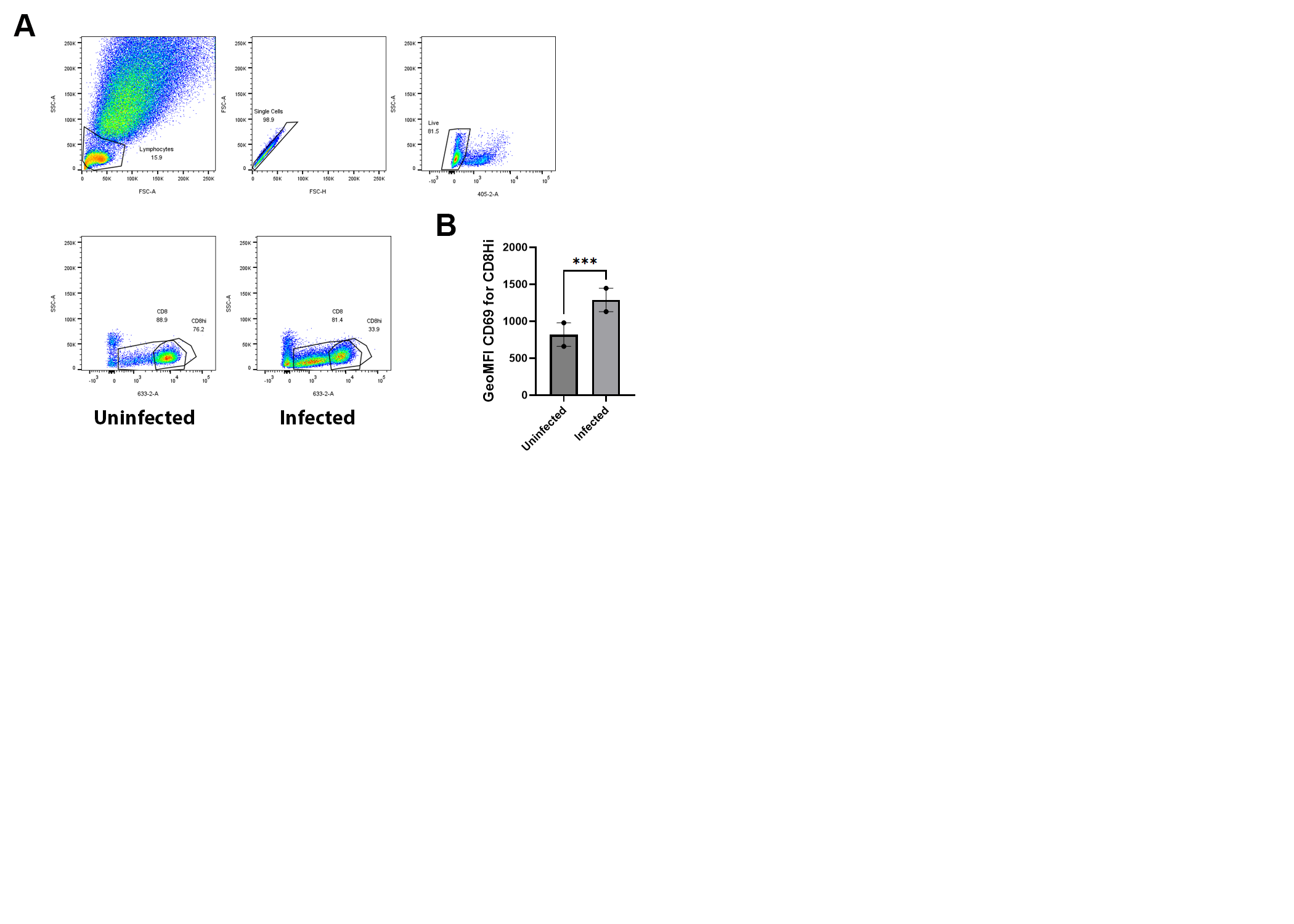

Supplement: S1 Fig — (A) Gating strategy on MAIT cells that are CD8 high. (B) CD69 gMFI pooled from two independent experiments. Significance measured by a paired two tailed t test. P = 0.0007. (TIF) [file ppat.1013342.s002.tif]

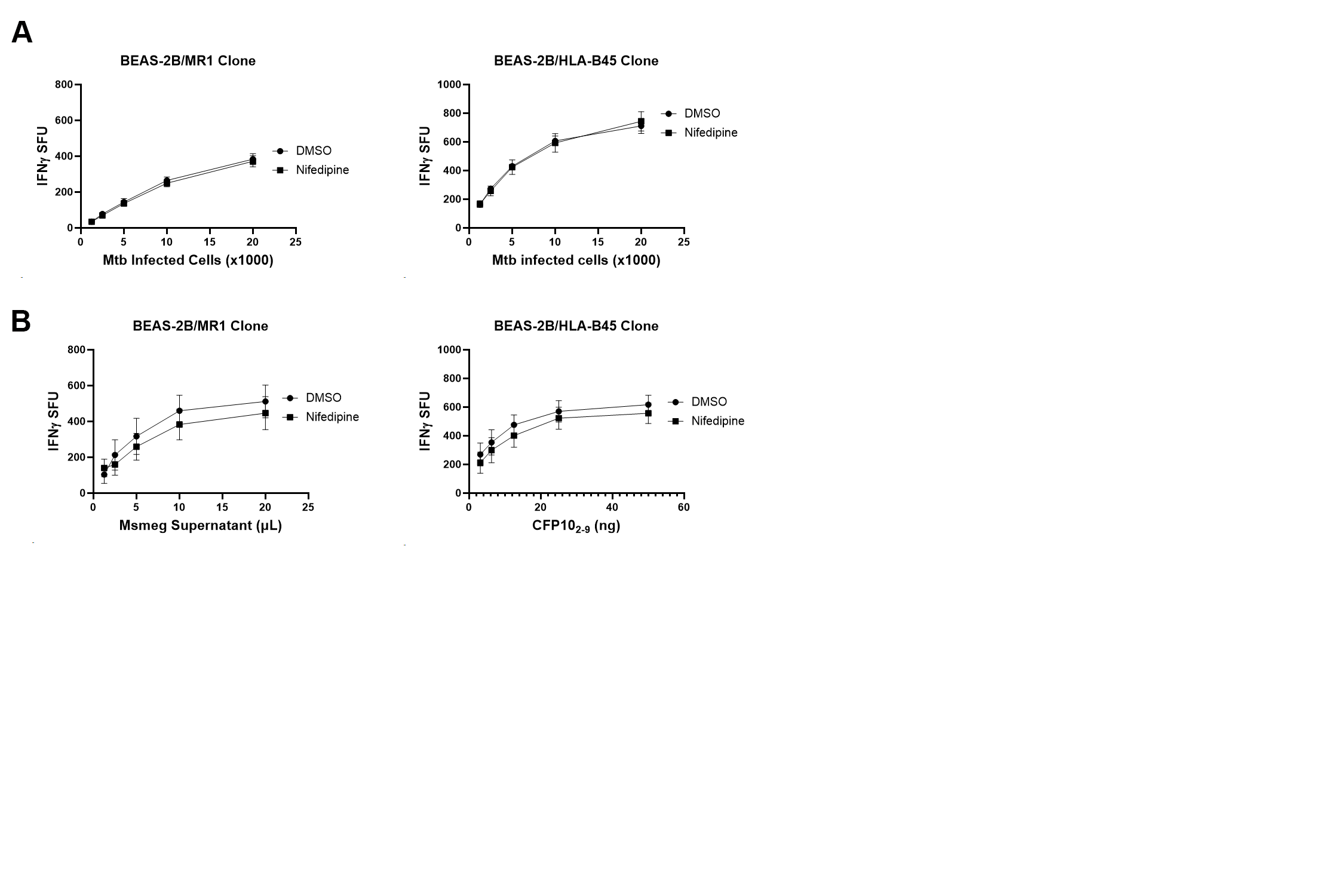

Supplement: S2 Fig — (A) IFN-γ ELISpot assays of BEAS-2B infected with Mtb and treated with 50µM Nifedipine or DMSO. Cells were incubated with MAITs (left) or HLA-B45-restricted (right) T cell clones. Mean values from technical replicates were pooled from 4 independent experiments (mean and SEM graphed). (B) IFN-γ ELISpot assays of effect of Nifedipine on MR1 and HLA-B45 presentation of exogenous antigens. Mean values from technical replicates were pooled from 4 independent experiments (mean and SEM graphed). (TIF) [file ppat.1013342.s003.tif]

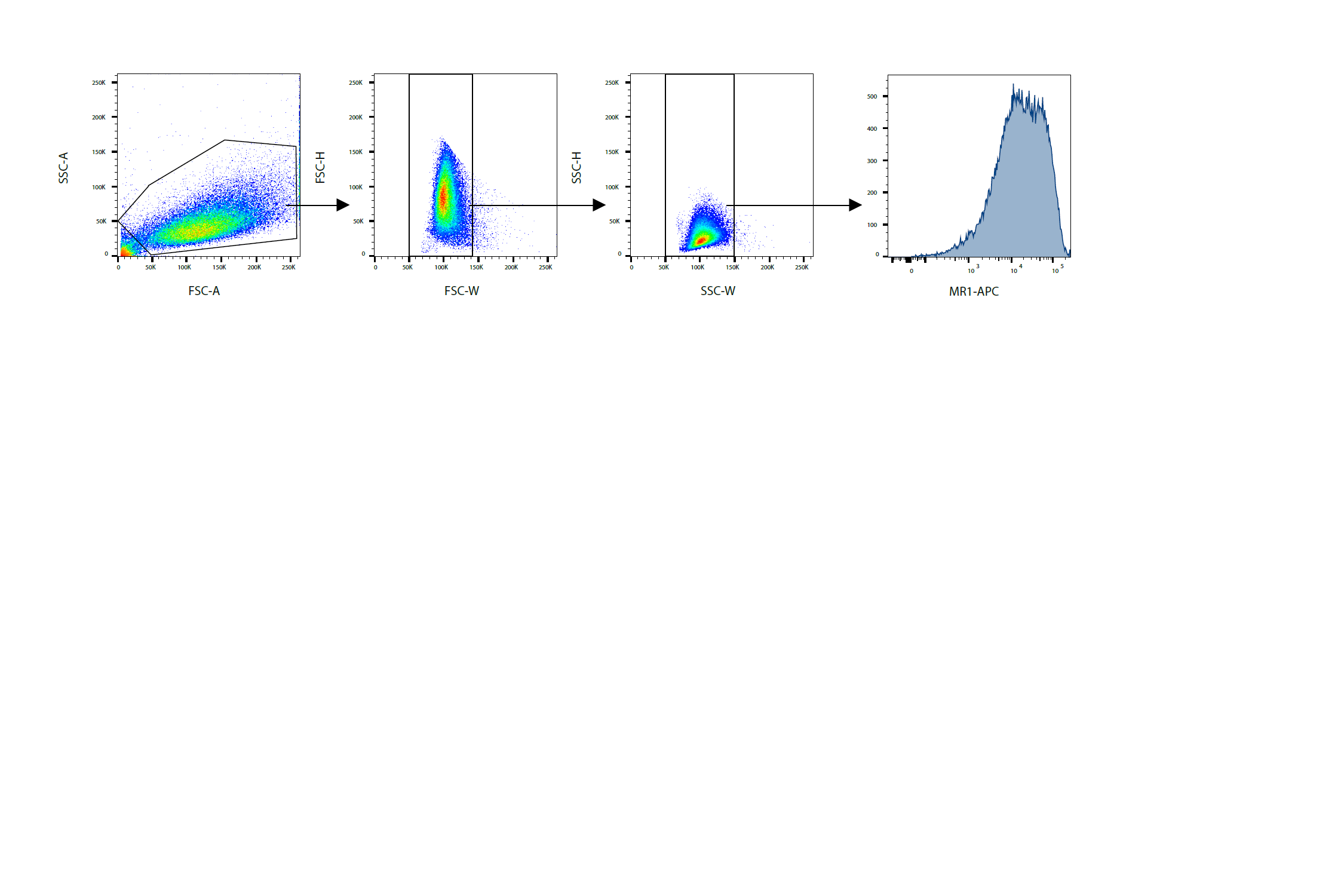

Supplement: S3 Fig — (TIF) [file ppat.1013342.s004.tif]

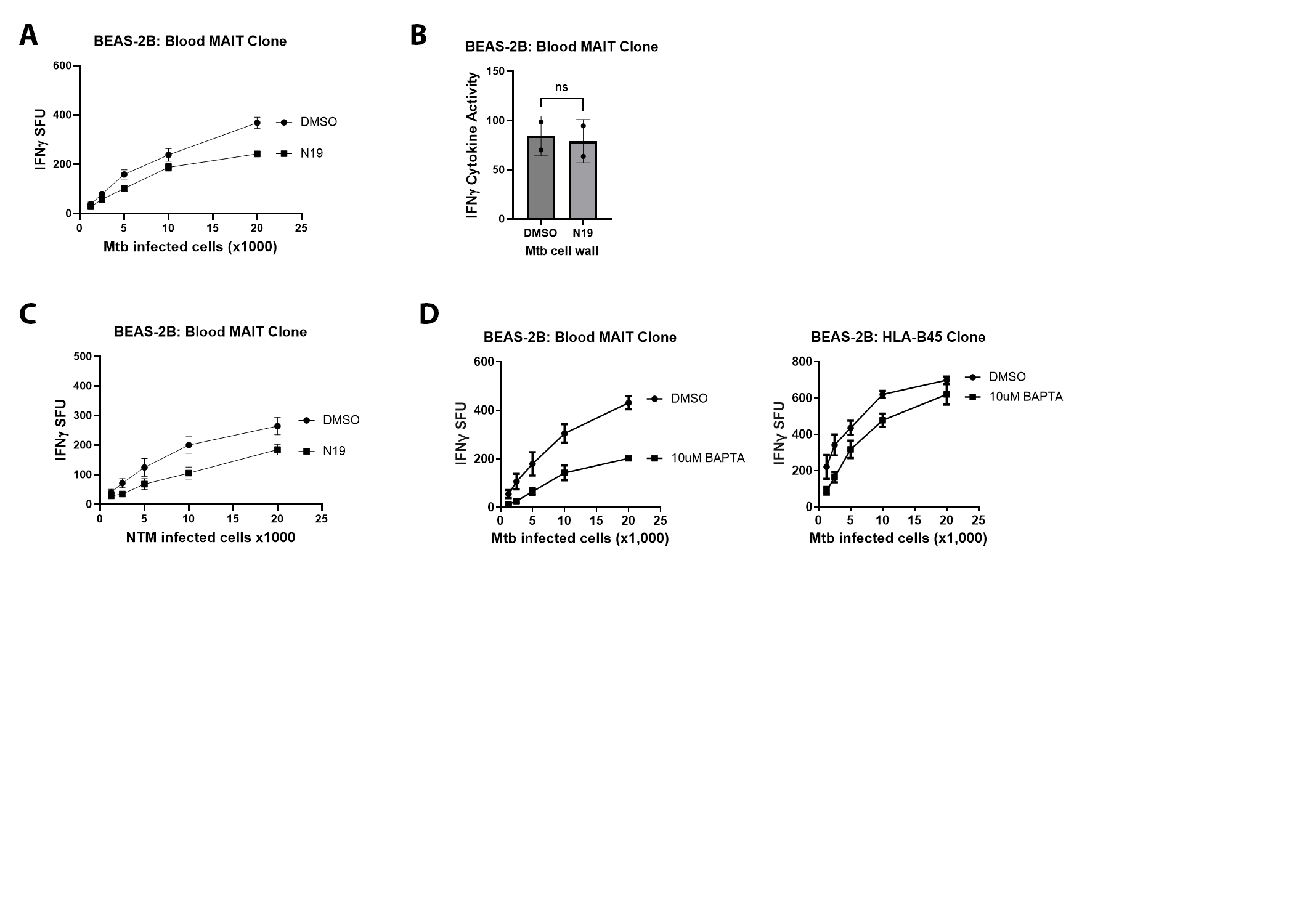

Supplement: S4 Fig — (A) N19 decreases MR1 presentation of Mtb when added at the same time as infection. 25µM N19 was added to BEAS-2Bs the same time as Mtb (MOI 8). The next day, the cells were incubated with a Blood MAIT clone as discussed in Fig 1. Pooled data from 3 independent experiments. P < 0.0001. (B) N19 has no effect on exogenously added Mtb cell wall. 25µg of Mtb cell wall was incubated with BEAS-2B treated with N19 versus DMSO and co-incubated with a Blood MAIT clone. Pooled data from 2 independent experiments. Significance measured by a paired two tailed t test. P = 0.15. (C) N19 decreases MR1 dependent presentation of nontuberculous mycobacteria (NTM). IFN-γ ELISpot assays of BEAS-2B infected with NTM (Mycobacterium avium Chester, ATCC) at an MOI of 22 and treated with 25µM N19 or DMSO. Cells were incubated with MAIT cell clones and IFN-γ spot forming units (SFU) measured. Mean values from technical replicates were pooled from 3 independent experiments (mean and SEM graphed). (D) Effect of the calcium chelator BAPTA-AM on MR1 presentation of Mtb. BEAS-2B were treated with 10µM BAPTA-AM versus DMSO. Mtb was added at the same time (MOI 8). Cells were incubated with a Blood MAIT clone versus an HLA-B45 restricted clone. Pooled data from 2 independent experiments. Left, P < 0.0001. Right, P = 0.0001. (TIF) [file ppat.1013342.s005.tif]

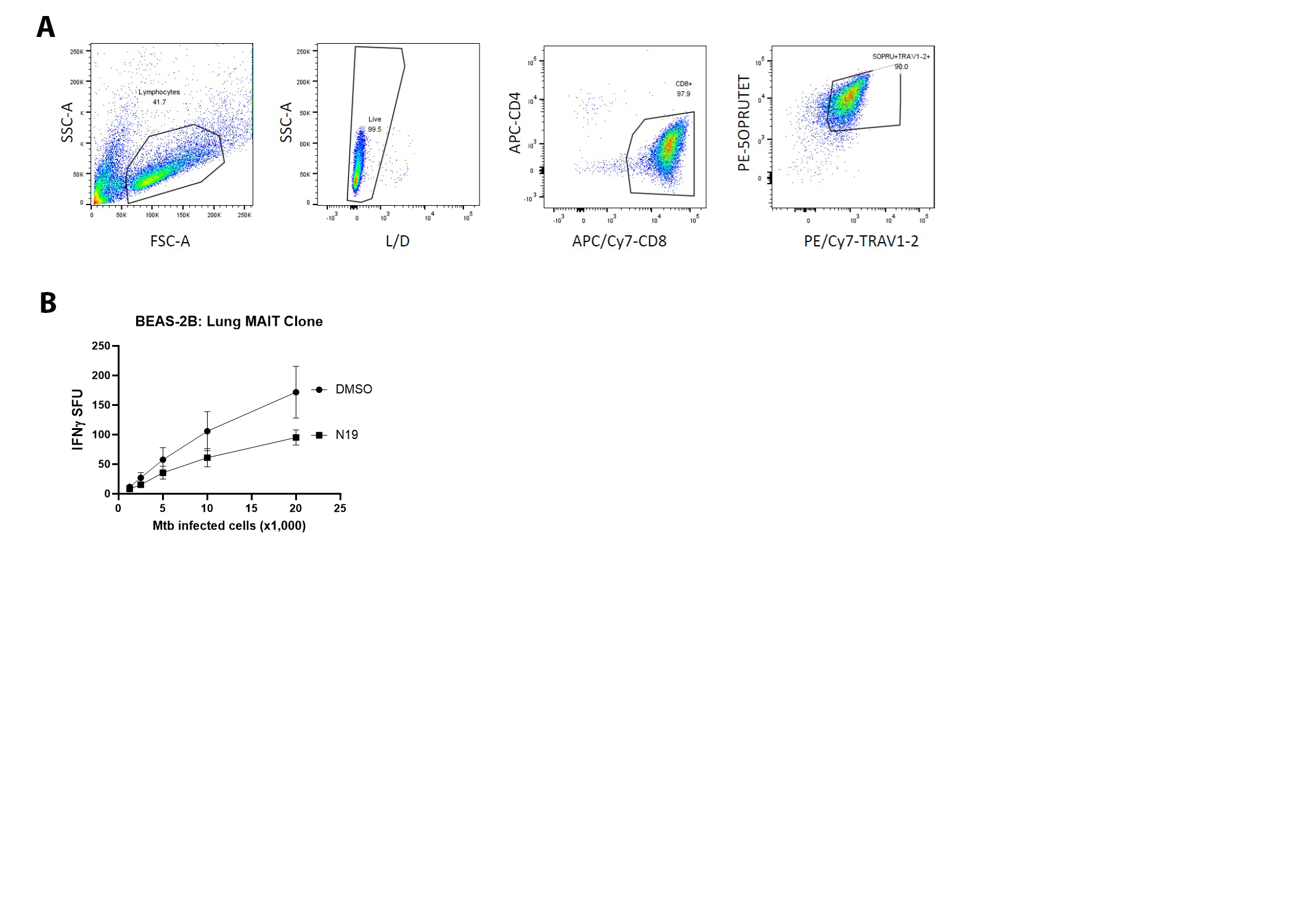

Supplement: S5 Fig — (A) Lung MAIT was isolated from BAL as described by Wong et al [33]. Flow cytometry characterizing Lung MAIT D1004-A3 as an MR1/5-OP-RU tetramer positive, TRAV1–2 + MAIT cell. (B) Effect of 25µM N19 versus DMSO on Mtb infected BEAS-2B incubating with 20,000 D1004-A3 clones. Pooled data from 3 independent experiments. P = 0.0043. (TIF) [file ppat.1013342.s006.tif]

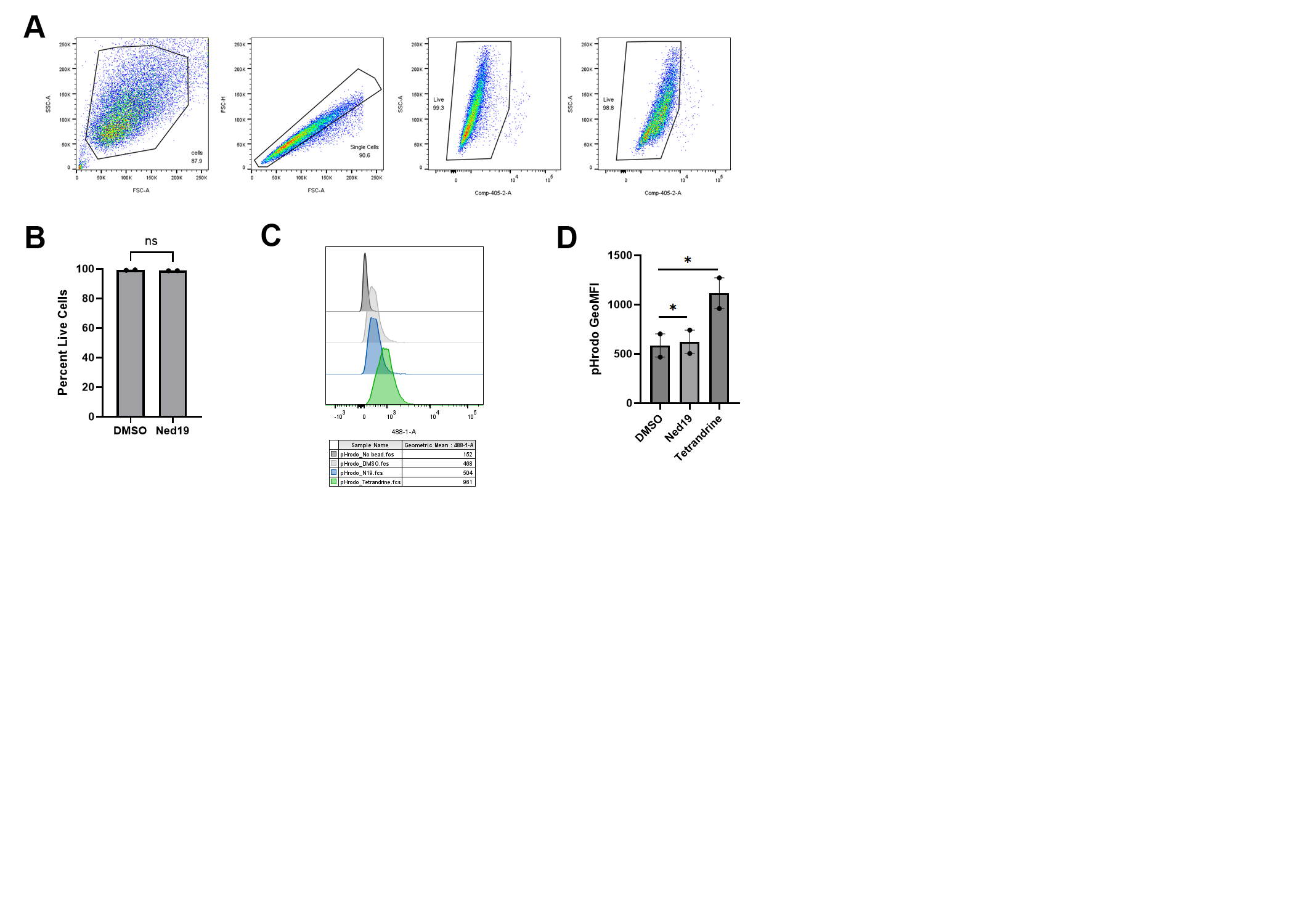

Supplement: S6 Fig — (A) Gating strategy for live dead staining of N19 and DMSO treated BEAS-2B. (B) Mean data plotted from technical replicates of live dead staining from 2 independent experiments. Significance measured with an unpaired t test. (C) Flow histogram of pHrodo dextran green of BEAS-2B treated with DMSO, N19 or tetrandrine. (D) Pooled data of pHrodo dextran green from 2 independent experiments. gMFI: DMSO = 585.5, N19 = 623.0, tetrandrine = 1117. Statistical significance measured with paired two tailed t test. (TIF) [file ppat.1013342.s007.tif]

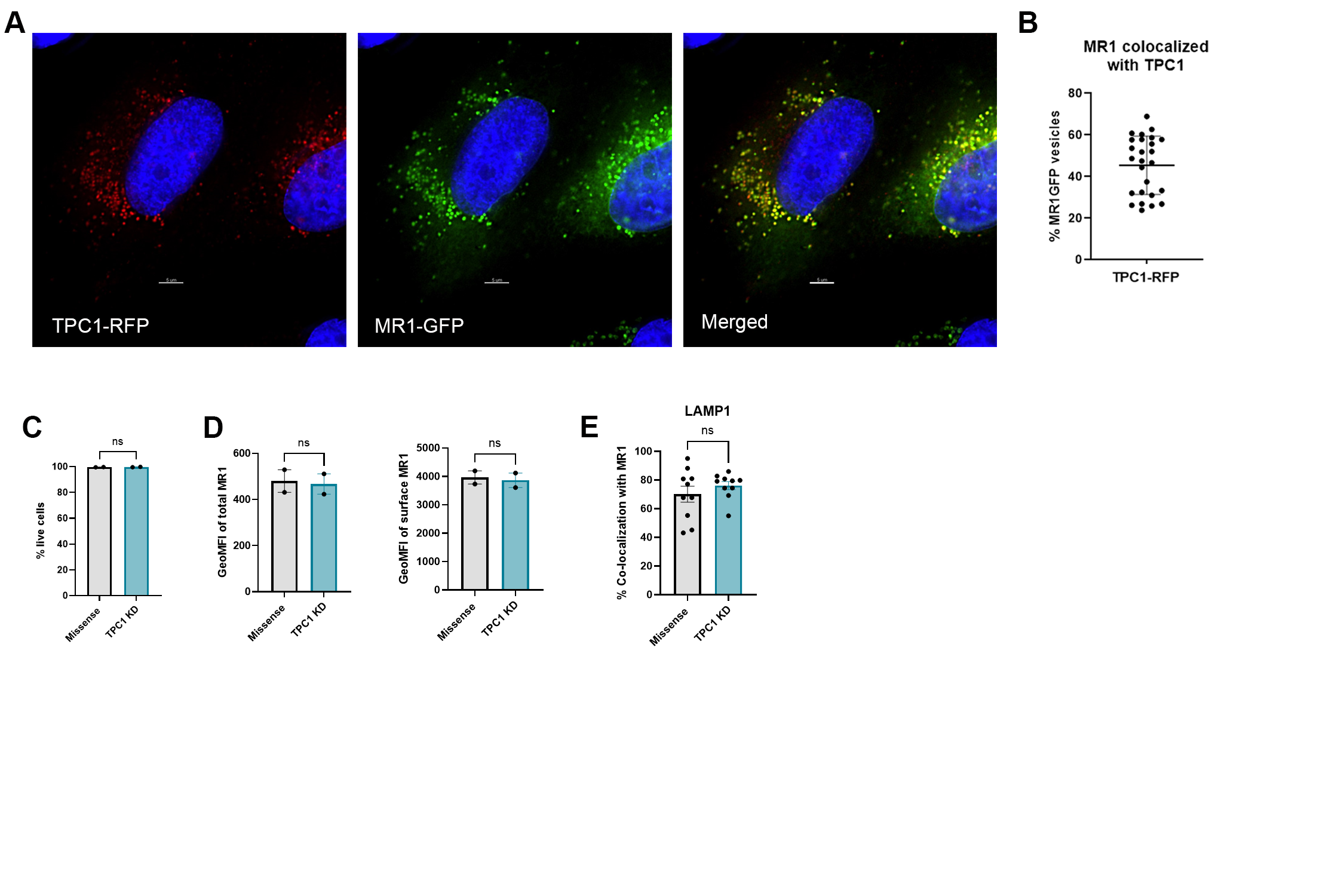

Supplement: S7 Fig — (A) Microscopy of MR1-GFP and TPC1-RFP. BEAS-2B expressing TET-MR1GFP transfected with TPC1-RFP. MR1GFP was induced with doxycycline and cells imaged the next day. Nuclei were stained with Hoechst 33342. Images are representative of 30 different images from 3 independent experiments. Scale bars represent 5µm. (B) Quantification of percent of MR1 vesicles that localized with TPC1. Data are graphed as mean and standard deviation. Mean percentage is 44% with a standard deviation of 14.5%. (C) Live dead staining of TPC1 knockdown in BEAS-2B. 2 independent experiments performed. Statistical analysis performed with a paired t test. (D) Total (left) and surface (right) MR1 in TET-MR1GFP cells with TPC1 knocked down. Analysis as performed in (C). (E) LAMP1 colocalization with MR1 in TET-MR1GFP cells with TPC1 knock down. 10 cells imaged in each condition. Statistical analysis done by an unpaired t test. (TIF) [file ppat.1013342.s008.tif]
